# Airway Management During Emergent Laparotomy in a Patient With Subglottic Stenosis and Unilateral True Vocal Cord Paralysis: A Case Report

**DOI:** 10.7759/cureus.88659

**Published:** 2025-07-24

**Authors:** Rayyan Bhutta, Ali Osman, Connor Henn, Hameed H Salah, Samir Patel

**Affiliations:** 1 Anesthesiology, Ohio University Heritage College of Osteopathic Medicine, Dublin, USA; 2 Anesthesiology and Perioperative Medicine, OhioHealth Doctors Hospital, Columbus, USA

**Keywords:** airway management, anesthesiology, endotracheal tube use, laryngoscopy and endotracheal intubation, subglottic stenosis, vocal cord paralysis

## Abstract

This case report describes the airway course of a 52-year-old male who required emergent exploratory laparotomy for suspected gastrointestinal perforation. The patient had underlying subglottic stenosis and unilateral vocal cord paralysis, as well as a history of significant cardiopulmonary disease. Attempts at intubation using standard tube sizes were unsuccessful. A 6.0 endotracheal tube was eventually passed beyond a point of resistance, allowing the operation to proceed. He remained intubated after surgery and was successfully extubated several days later. The report emphasizes the need for adaptable airway plans and highlights the constraints that may arise during urgent operative care.

## Introduction

Upper airway narrowing due to conditions such as subglottic stenosis or vocal cord immobility can increase the risk of intubation failure, especially during procedures where time constraints preclude a full preoperative workup [1]. These structural issues often go unrecognized until induction, particularly in patients without prior airway assessment [2]. In patients with reduced cardiopulmonary reserve, even brief airway complications may carry an elevated risk [3]. Subglottic stenosis is a condition that can present with a variety of symptoms, ranging from dyspnea, stridor, or hoarseness to life-threatening airway compromise [4]. Similarly, vocal cord paralysis can result in symptoms of stridor, coughing, and obstructive symptoms [5]. When these conditions coexist, they may create an extremely difficult anatomical airway to intubate, causing complications during induction. This report illustrates the significance of anticipating difficult airway situations even in the absence of formal evaluation.

## Case presentation

**Figure 1:**
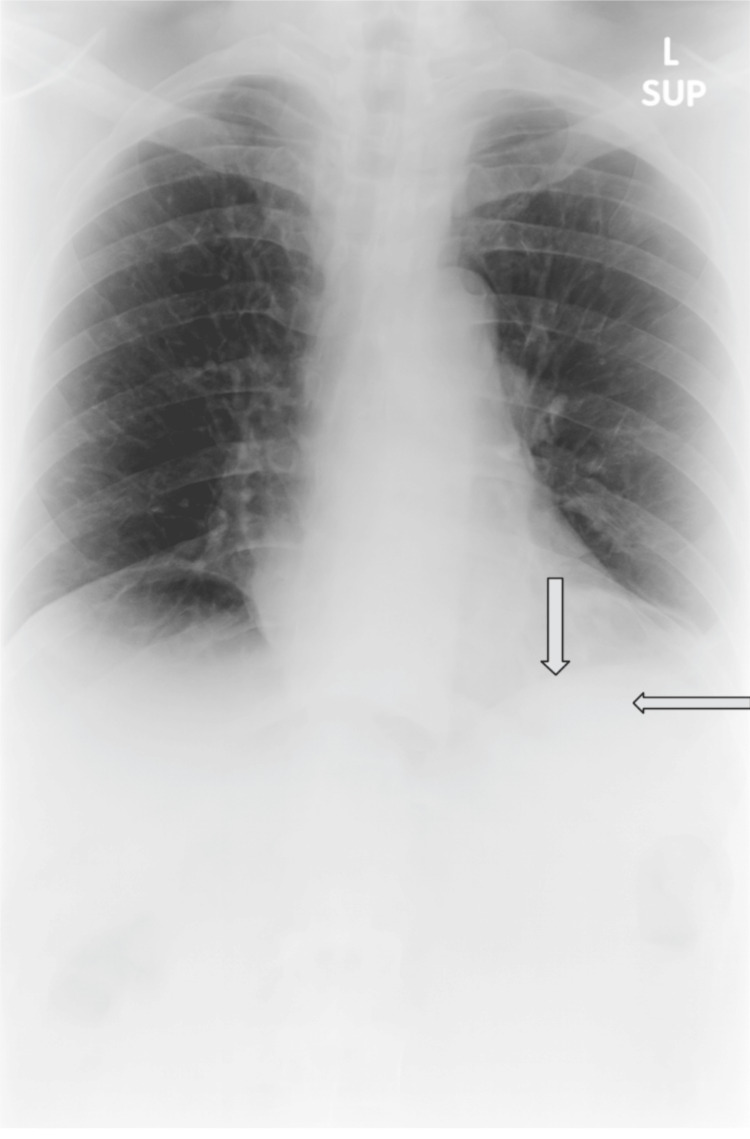
Chest X-ray of the patient revealing signs of consolidation of the left lower lobe, consistent with lobar pneumonia

A 52-year-old male was admitted for the management of a chronic obstructive pulmonary disease (COPD) exacerbation with associated left lower lobe pneumonia and hoarseness (Figure 1).

Significant past medical history included metastatic prostate cancer, pulmonary hypertension, COPD, right heart failure, left vocal cord paralysis, and tracheostomy. His medications included bronchodilators, corticosteroids, neuropathic agents, antidepressants, and opioids at home.

**Figure 2:**
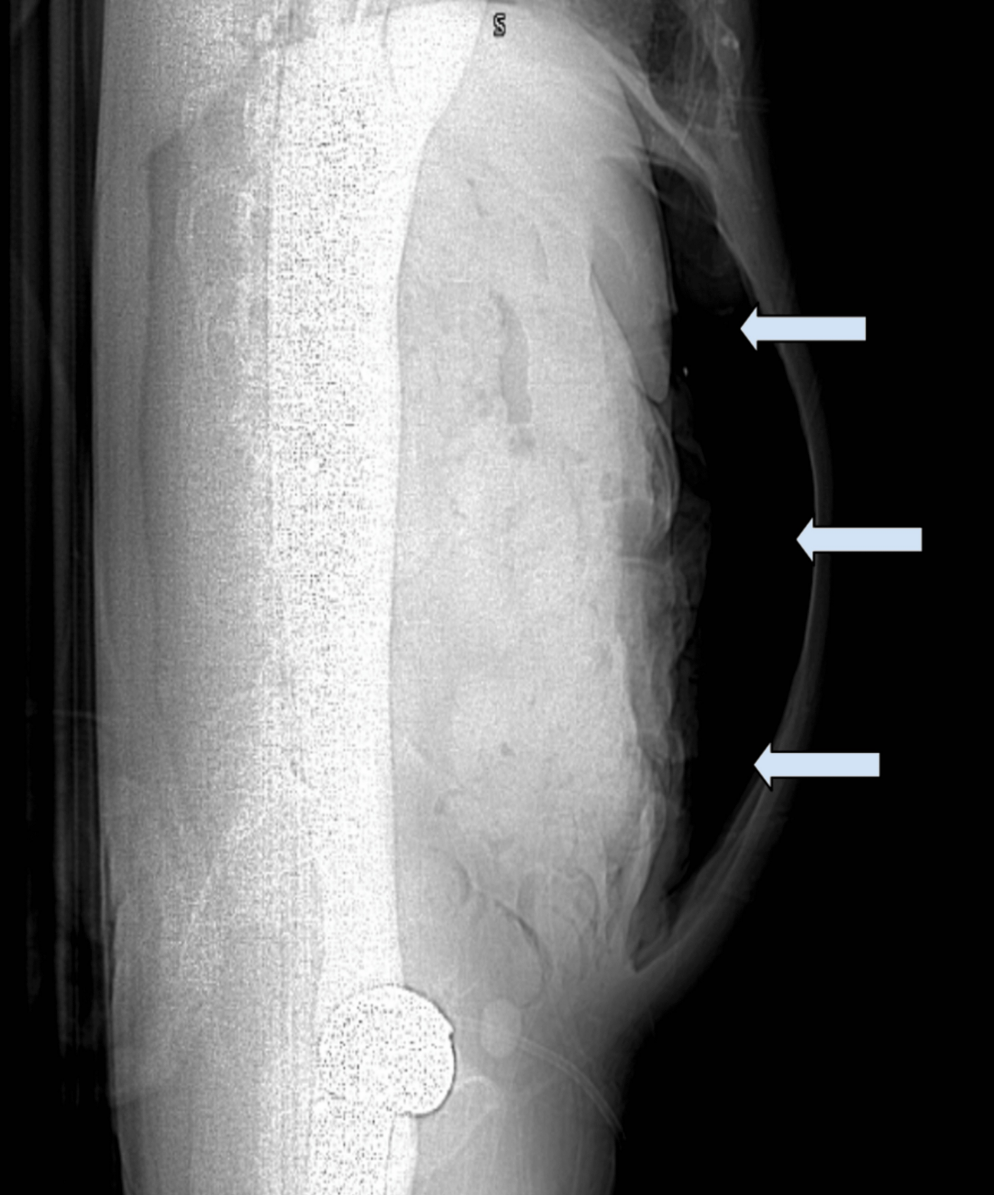
Sagittal abdominal X-ray of the patient showing signs of pneumoperitoneum, which prompted the emergent exploratory laparotomy

During the patient's hospital stay, he developed worsening abdominal distension and new-onset pain. Imaging with plain film and CT confirmed free intraperitoneal air consistent with a perforated viscus (Figure 2). Emergent exploratory laparotomy was recommended, requiring surgical intervention within two hours. Due to limited time, only a focused perioperative evaluation was completed. Prior documentation mentioned possible subglottic narrowing and vocal cord dysfunction, but no laryngoscopic or updated radiographic airway assessment was available to visualize the extent of airway stenosis.

Transthoracic echocardiography showed right ventricular strain with septal flattening, in addition to pulmonary hypertension. The patient was classified as ASA IV. Arterial line placement was planned before induction, followed by central line placement once the airway was secured. The patient was counseled about anesthesia risks and the potential for a repeat tracheostomy. Verbal consent was obtained.

**Figure 3:**
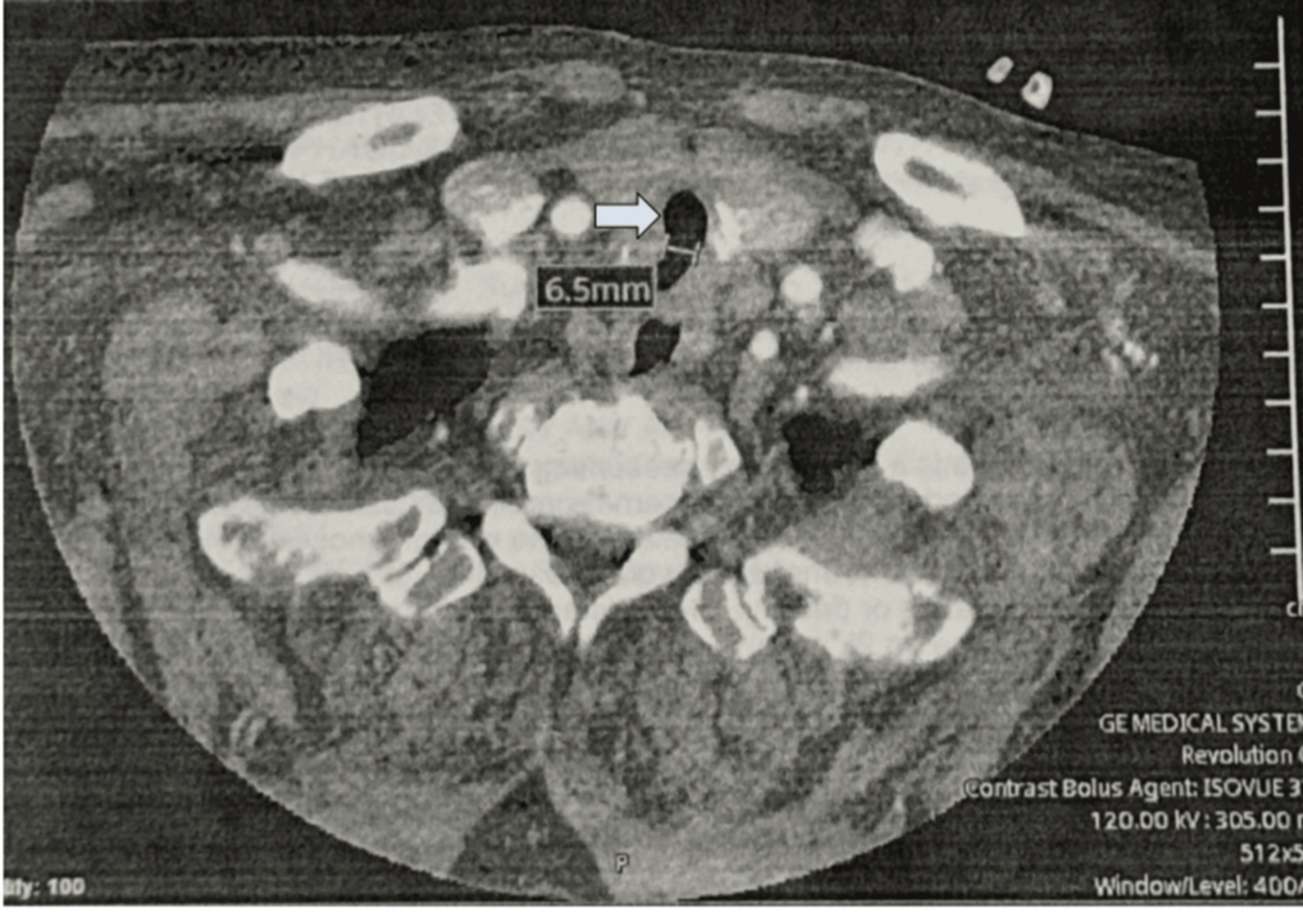
CTA of the patient's head and neck from 2024 revealing subglottic stenosis CTA: computed tomography angiography

An 8.0 mm internal diameter endotracheal tube was initially chosen to permit bronchoscope passage if needed. Although the vocal cords were visualized clearly with video laryngoscopy, the tube was obstructed before entering the trachea. A 7.0 tube passed through the glottis but encountered subglottic resistance, preventing advancement for sufficient cuff inflation (Figure 3). A 6.0 tube was then inserted and advanced without resistance. Position was confirmed with end-tidal CO₂, bilateral breath sounds, and symmetric chest movement.

Vasoactive agents such as vasopressin, norepinephrine, and epinephrine were prepared before induction and administered as needed. The surgery proceeded without further complications. The patient remained intubated and was transferred to the ICU for postoperative monitoring.

The ICU team was informed about the difficult airway and advised to proceed cautiously with extubation. The patient was monitored for cuff leak and ventilatory parameters. He was maintained on ventilatory support, and corticosteroids were administered to reduce airway edema. After several days, he completed spontaneous breathing trials without any evidence of obstruction. He was extubated successfully and transitioned to a nasal cannula. He was later discharged in stable condition.

## Discussion

Managing the airway in patients with subglottic narrowing often calls for intraoperative adjustments [1]. In elective settings, airway imaging or endoscopic evaluation may guide preoperative planning and help identify anatomical risks [2]. Urgent surgeries, by contrast, are often approached with targeted management to maintain oxygenation, advanced intubation techniques, and teamwork-based care [6]. In this case, an 8.0 tube was initially selected, but subglottic resistance necessitated a smaller 6.0 tube to maintain ventilation. Had the 6.0 ETT also failed, the next steps in this specific case would have involved the use of a supraglottic airway device such as a laryngeal mask airway (LMA). However, it is important to note that while LMAs can be used in difficult airway situations, they do not offer protection against aspiration [7]. The LMA could potentially pose a significant threat to the patient if used long-term, as the patient had not completed the recommended fasting period before general anesthesia, given the emergent nature of this case

Subglottic stenosis and unilateral vocal cord paralysis may both restrict airway diameter. When combined, these conditions increase the likelihood of partial or failed intubation. Early signs such as hoarseness or stridor may be present but are often nonspecific [8]. Preoperative screening for airway compromise remains inconsistent outside of elective surgical pathways. Documentation of airway history and tube sizing should be included in patient records to guide future care. In addition to the anatomical challenges, our patient presented with pulmonary hypertension and right heart strain, both of which raise the perioperative risk of mortality [3]. Vasoactive agents were prepared in advance to avoid intraoperative hypotension. Anticipatory hemodynamic support and invasive monitoring helped maintain stability.

## Conclusions

This report illustrates the importance of flexible airway planning in time-constrained operative scenarios. Subglottic stenosis and vocal fold immobility may present without prior diagnosis and complicate routine intubation approaches. When elective evaluation is not possible, intraoperative adaptability and clear postoperative communication become critical to patient safety.
